# Decolorization of synthetic textile wastewater using electrochemical cell divided by cellulosic separator

**DOI:** 10.1186/s40201-017-0273-3

**Published:** 2017-05-25

**Authors:** Ali Asghar Najafpoor, Mojtaba Davoudi, Elham Rahmanpour Salmani

**Affiliations:** 1 Health Sciences Research Center, Department of Environmental Health Engineering, School of Health, Mashhad University of Medical Sciences, Mashhad, Iran; 2 Department of Environmental Health Engineering, School of Health, Torbat Heydariyeh University of Medical Sciences, Torbat Heydariyeh, Iran; 3 Student Research Committee, School of Health, Mashhad University of Medical Sciences, Mashhad, Iran

**Keywords:** Cellulosic separator, Electro-oxidation, Electro-reduction, Graphite anodes, Reactive red 120

## Abstract

**Background:**

Annually, large quantities of dyes are produced and consumed in different industries. The discharge of highly colored textile effluents to the aquatic environments causes serious health problems in living organisms. This paper investigates the performance of each of the electro-oxidation and electro-reduction pathways in the removal of reactive red 120 (RR120) from synthetic textile effluents using a novel electrochemical reactor.

**Methods:**

In the current study, a two-compartment reactor divided by cellulosic separator was applied in batch mode using graphite anodes and stainless steel cathodes. Central Composite Design was used to design the experiments and find the optimal conditions. The operational parameters were initial dye concentration (100–500 mg L^−1^), sodium chloride concentration (2500–12,500 mg L^−1^), electrolysis time (7.5–37.5 min), and current intensity (0.06–0.3 A).

**Results:**

The results showed that electro-oxidation was much more efficient than electro-reduction in the removal of RR120. According to the developed models, current intensity was the most effective factor on the electro-oxidation of RR120 as well as in power consumption (Coefficients of 12.06 and 0.73, respectively). With regard to the dye removal through electro-reduction, electrolysis time (coefficient of 8.05) was the most influential factor. Under optimal conditions (RR120 = 200 mg.L^−1^, NaCl = 7914.29 mg.L^−1^, current intensity = 0.12 A, and reaction time = 30 min), the dye was removed as 99.44 and 32.38% via electro-oxidation and electro-reduction mechanisms, respectively, with consuming only 1.21 kwhm^−3^ of electrical energy.

**Conclusions:**

According to the results, electro-oxidation using graphite anodes in a cell divided by cellulosic separator is very efficient, compared to electro-reduction, in the removal of RR120 from aqueous solutions.

## Background

Wastewater generation in huge volumes is one of the consequences of uncontrolled demand for textile articles, which causes extreme water consumption by textile industries [1]. Different wet-processing operations in the manufacturing process of textile industry result in the production of effluent which contains various pollutants including dyes, surfactants, detergents, and suspended solids [2]. Azo dyes as the largest group of organic dyes [3] constitute 20–40% of the dyes used in the textile industry [4] and are the most frequent chemical class of dyes applied to industrial scale [5]. The general chemical formula of azo compounds has been shown in the form of R-N = N-R functional group. In the structure of these compounds, the double bond between nitrogen atoms indicates the azo chromophores, while R is the aromatic ring [1] containing groups such as sulfonate and hydroxyl [3]. The relatively low degree of dye fixation to fabrics especially for the reactive dyes results in the release of unfixed dyes into the effluent [6, 7]. It has been stated that textile industries produce a strongly colored wastewater [8]. It has also been declared that even the presence of inconsiderable dye concentrations in the effluent can reduce the penetration of light into the receiving water bodies. This leads to devastating effects on the aquatic biota [9] such as photosynthetic activity of aquatic plants [8]. The probable persistence and the long-term bioaccumulation of synthetic organic dyes severely damage the health of ecosystems and living organisms [10].

A wide range of technologies for the removal of dyes from contaminated effluents can be found in literature [11]. Conventional treatment methods, i.e., physical, chemical, and biological processes, are still highly used. The physical methods mainly are practical for separating the solid pollutants, since there must be a difference between the pollutant and its media regarding the physical property. It is noticeable that chemical treatment occurs just under conditions that electrostatic property of both pollutant and coagulant is compatible [12]. Undesirable efficiency, high cost, and secondary pollutants are major shortcomings of physicochemical processes [9]. In spite of the fact that synthetic dyes have properties such as stability against light, temperature, and biodegradability [4], which makes decolorization difficult and incomplete [6], it was stated by Kariyajjanavar et al. that azo dyes are non-resistant to biological treatment methods under anaerobic conditions. However, applying this method is not suggested for dye removal as the products resulted from breakdown of azo dyes can be more toxic than the dye molecules [4]. The adverse environmental and health effects of dyes and their degradation products have pushed scholars’ efforts towards developing powerful and effective treatment technologies [13]. According to the literature review, numerous advanced methods including adsorption, biosorption, reverse osmosis, ion-exchange [6], membrane separation, electro kinetic coagulation, irradiation, ozonation [8], sonication, enzymatic treatments, engineered wetland systems [9], and advanced oxidation processes (AOPs) such as TiO_2_ photo-catalysis and electrochemical methods [10] have been utilized by researchers for the efficient treatment of textile wastewater. The electrochemical advanced oxidation processes (EAOPs) have received special interest for water and wastewater remediation [13]. Among them, electrocoagulation (EC) [14], electro-oxidation (EO) [15], and electro-Fenton (EF) [16] have been frequently studied. EAOPs have some significant advantages such as simple equipment [4], easy implementation [17], close control of the favored reactions through applying optimum electrical current, on-site treatment in less space [18], and high efficiency for the degradation of persistent pollutants, while the cost of electricity used can be a drawback [19]. The presence of iron ions in the EC and EF processes leads to the sludge generation that imposes the cost of further treatment [20]. EO is the most widely used mechanism of EAOPs [21] and anodic oxidation (AO) is the most typical kind of EO [22]. The explanation of complicated electrochemical reactions that occur during the EO treatment process and determining the definite removal mechanism of many of the contaminants do not seem an easy task [6]. EO of pollutants can occur through AO directly or indirectly, and also by the participation of chlorine-based oxidants when chloride solutions are treated [18]. In direct AO, pollutant molecules are oxidized at anode via electron transfer from the organic matter to the electrode, while in the indirect AO, the chemical reactions with electro-generated species such as hydroxyl radicals resulted from water discharge at the anode leads to the pollutant degradation [23]. It is known that direct AO leads to poor decontamination, while the effectiveness of indirect AO is dramatically dependent on the used anode. In the so-called “active” anodes which have low oxidation power, the chemisorbed “active oxygen” (MO_x+1_) is the yield of water oxidation, while the physisorbed hydroxyl radical is the product of water discharge at the high oxidation power anodes also named “non-active”. The Pt, IrO_2_, and RuO_2_ are some examples of the former anodes in the formation of selective oxidation products (Eq. 1), while boron-doped diamond (BDD), PbO_2_, and SnO_2_ are typical kinds of the latter anodes causing complete combustion of the organic compounds (R) (Eq. 2) [24]:

1 $$ \mathrm{R} + {\mathrm{MO}}_{\mathrm{X}+1}\to\ \mathrm{R}\mathrm{O} + {\mathrm{MO}}_{\mathrm{X}} $$

2 $$ \mathrm{R} + {\mathrm{MO}}_{\mathrm{X}}\left(\bullet \mathrm{OH}\right)\ \to\ {\mathrm{CO}}_2 + {\mathrm{H}}^{+} + \mathrm{e} + {\mathrm{MO}}_{\mathrm{X}} $$

Although non- active anodes have been preferred in most of the EO studies for their ability in quick and total mineralization, it was shown by Méndez-Martínez et al. [10] that the use of active anodes can be equally interesting as they provide the chance for thorough elucidation of the general degradation mechanisms.

As previously stated, in chloride medium, the oxidation of organics can also occur by chlorine-based oxidants. The presence of NaCl in the reaction mixture leads to the formation of chlorohydroxyl radicals (ClOH^•^) on the anode surface (Eq. 3), which oxidize the organic matter as given in Eq. (4):

3 $$ {\mathrm{H}}_2\mathrm{O} + \mathrm{M} + {\mathrm{Cl}}^{\hbox{-}}\to\ \mathrm{M}\left[{\mathrm{Cl}\mathrm{OH}}^{\bullet}\right] + {\mathrm{H}}^{+} + 2{\mathrm{e}}^{\hbox{-} } $$

4 $$ \mathrm{R} + \mathrm{M}\left[{\mathrm{Cl}\mathrm{O}\mathrm{H}}^{\bullet}\right]\ \to\ \mathrm{M} + \mathrm{R}\mathrm{O} + {\mathrm{H}}^{+} + {\mathrm{Cl}}^{\hbox{-} } $$

Furthermore, primary oxidants such as oxygen (Eq. 5), chlorine (Eq. 6), hydrogen peroxide (Eq. 7), and hypochlorite (Eq. 8) can result from the reactions between water and radicals near the anode:

5 $$ {\mathrm{H}}_2\mathrm{O} + \left[{\mathrm{MOH}}^{\bullet}\right]\ \to\ \mathrm{M} + {\mathrm{O}}_2 + 3{\mathrm{H}}^{+} + 3{\mathrm{e}}^{\hbox{-} } $$

6 $$ {\mathrm{H}}_2\mathrm{O} + \mathrm{M}\left[{\mathrm{Cl}\mathrm{OH}}^{\bullet}\right] + {\mathrm{Cl}}^{\hbox{-}}\to\ \mathrm{M} + {\mathrm{O}}_2 + {\mathrm{Cl}}_2 + 3{\mathrm{H}}^{+} + 4{\mathrm{e}}^{\hbox{-} } $$

7 $$ {\mathrm{H}}_2\mathrm{O} + \left[{\mathrm{MOH}}^{\bullet}\right]\ \to\ \mathrm{M} + {\mathrm{H}}_2{\mathrm{O}}_2 + {\mathrm{H}}^{+} + {\mathrm{e}}^{\hbox{-} } $$

8 $$ {\mathrm{H}}_2\mathrm{O} + {\mathrm{Cl}}^{\hbox{-}}\to\ \mathrm{HOCl}\ \left(\mathrm{HOCl}\leftrightarrow\ {\mathrm{Cl}\mathrm{O}}^{\hbox{-} } + {\mathrm{H}}^{+}\right) + {\mathrm{H}}^{+} + 2{\mathrm{e}}^{\hbox{-} } $$

Then, the reaction between free chlorine and oxygen results in the formation of secondary oxidants such as chlorine dioxide and ozone according to the reactions presented by Eq. (9) and Eq. (10) [7]:

9 $$ {\mathrm{H}}_2\mathrm{O} + \mathrm{M}\left[{\mathrm{Cl}\mathrm{O}\mathrm{H}}^{\bullet}\right] + {\mathrm{Cl}}_2\to\ \mathrm{M} + {\mathrm{Cl}\mathrm{O}}_2 + 3{\mathrm{H}}^{+} + 2{\mathrm{Cl}}^{\hbox{-} } + {\mathrm{e}}^{\hbox{-} } $$

10 $$ {\mathrm{O}}_2 + \mathrm{M}\left[{\mathrm{O}\mathrm{H}}^{\bullet}\right]\ \to\ \mathrm{M} + {\mathrm{O}}_3 + {\mathrm{H}}^{+} + {\mathrm{e}}^{\hbox{-} } $$

Simultaneously with the oxidation reactions at the anodic chamber, the reduction reactions occur in the cathodic compartment. Based on the scientific evidence, the electrochemical reduction, or in other words, the electro-reduction (ER) method has been applied for the removal of dyes and many of the other contaminants from both synthetic and real effluents [20]. However, a limited number of studies have tried ER compared to AO mainly because of the performance dissatisfaction [24]. ER was considered by Bechtold et al. as a proper method for the treatment of strongly colored effluents containing reactive dyes. They remarked that partial reduction of dye (Eq. 11) produces hydrazine, while its total reduction generates the amino compounds (Eq. 12) [25]:

11 $$ {\mathrm{R}}_1\hbox{-} \mathrm{N}=\mathrm{N}\hbox{-} {\mathrm{R}}_2\to\ {\mathrm{R}}_1\hbox{-} \mathrm{N}\mathrm{H}\hbox{-} \mathrm{N}\mathrm{H}\hbox{-} {\mathrm{R}}_2 $$

12 $$ {\mathrm{R}}_1\hbox{-} \mathrm{N}\mathrm{H}\hbox{-} \mathrm{N}\mathrm{H}\hbox{-} {\mathrm{R}}_2\to\ {\mathrm{R}}_1{\mathrm{NH}}_2 + {\mathrm{R}}_2{\mathrm{NH}}_2 $$

Electrolytic hydrogenation is the most common cathodic reaction resulted from water electrolysis at the cathode surface [26] (Eq. 13 [2]):

13 $$ 2{\mathrm{H}}_2\mathrm{O} + 2{\mathrm{e}}^{\hbox{-}}\to\ {\mathrm{H}}_2 + 2{\mathrm{OH}}^{\hbox{-} } $$

Roessler et al. [27] have shown that the formed hydrogen can then react with the dye adsorbed at the cathode surface.

Radha et al. [2] achieved color removal efficiency equal to 96% via EO method in a batch system using stainless steel and graphite as cathode and anode, respectively, under 60 min reaction time at 0.6 A current intensity. Wang et al. [18] investigated the simultaneous removal of color and COD from real textile wastewater at the presence of Pt/Ti anode and graphite cathode in a divided reactor. Hypochlorite and hydrogen peroxide were determined as the main factors responsible for treatment process. The overall removal efficiency was proportional to the applied current and the color removal efficiency in the anodic chamber was much higher than cathodic one. Del Rio et al. [28] studied the effect of oxidation, reduction and oxido-reduction processes in divided and undivided electrolytic cells for the removal of Reactive Orange 4 dye. In the separated cell, a cationic membrane was used and the electrodes of Ti/SnO2–Pt–Sb and stainless steel were employed as anode and cathode, respectively. The maximum dye removal efficiency was obtained in the undivided cell via oxido-reduction reactions. Maljaei et al. [1] explored the removal of Reactive Yellow 3 dye through indirect EO method using graphite as both anode and cathode in a batch reactor. They reported higher efficiency of dye removal with increasing current intensity and decreasing electrolysis time. Kariyajjanavar et al. [4] introduced graphite as a relatively cheap anode, which provides satisfactory results for the electrochemical dye degradation.

The current study aimed to evaluate the performance of EO and ER using graphite anodes and stainless steel cathodes in the removal of RR120 dye from synthetic textile wastewater. We used a novel two-compartment reactor divided by cellulosic separator, while most of the electrochemical decontamination studies had been conducted in undivided cells. It is known that high cost of separation and non-ecofriendly properties of common membranes have restricted the use of divided cells. But interesting advantages of divided cells including the increased rate of electrochemical oxidation and reduction reactions, decreased generation of toxic by-products, and increased life-time of anode caused by acidic pH of the anodic compartment [26] encouraged the researchers of the current study to apply a low-cost material to divide the reactor to enjoy the benefits of divided cells. Therefore, cellulosic separator that was previously employed by Davoudi et al. [20] for synthetic tannery wastewater treatment was used in this project for synthetic textile wastewater treatment.

## Methods

### Chemicals

**Fig. 1:**
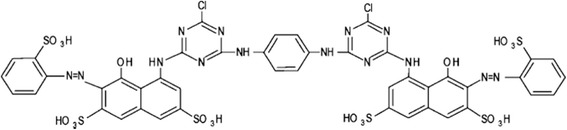
The molecular structure of RR120 dye

Double Distilled Water (DDW) was used to prepare stock solutions and required dilutions. Graphite electrode was purchased from Noavaran Shimi Company, Iran. Ultrapure grade of NaCl, sulfuric acid (H_2_SO_4_) and sodium hydroxide (NaOH) was obtained from Merck, Germany. To prepare 1000 ppm standard solution, 1 g of RR120 was weighed by a digital balance (Sartorius bp 110 s) and dissolved in 1000 mL of DDW. RR120 (red HE3B) was provided by Shadilon Textile Group Co., Iran. The molecular structure of the dye is demonstrated in Fig. 1 [29] and its physicochemical properties are shown in Table 1.

**Table 1:** The physicochemical properties of RR120

Chemical formula	C_44_H_24_Cl_2_N_14_Na_6_O_20_S_6_
CAS Registry Number	61951-82-4
Natural state	Powdered
Chemical structure	Diazo
Solubility in 20 °C water (g/l)	100
Molecular weight (g/mol)	1469.98
Charge	Negative
pH	6–9
Density (kg/m^3^)	450–500
λ_max_(nm)	530

### Experimental design

Experiments were designed based on CCD, a well-known design of Response Surface Methodology (RSM) in the Design Expert 7.0 (trial version). RSM is an efficient tool to optimize experimental conditions while minimizes the number of experiments [30]. Accordingly, 30 experiments were determined based on 2^4^ factorial designs with 6 central and 8 axial points. This number was obtained by defining the actual values of independent variables in the initial (−α) and end (+α) points by user while the three other levels of each parameter were suggested by CCD. Table 2 represents the real and coded values of operational factors. The expected responses in the current study were dye removal efficiency in each of the reactor chambers and consumed energy. In RSM, the experimental data corresponding to each dependent variable were fitted to a polynomial model to find the most influential factors and their various effects including linear, interaction, and quadratic effects. ANOVA was used to validate the adequacy of the models. To assess the quality of fit in the developed regression models, the coefficient of determination (R^2^) and adjusted R^2^ were applied. The Fisher distribution test (F-test) and adequate precision ratio were used to determine the statistical significance of the models and its associated terms [31].

**Table 2:** Independent variables in coded and real levels

Independent variables	Study levels				
	-α	−1	0	+1	+α
X_1_: RR120 Conc. (mg. L^−1^)	100	200	300	400	500
*X*_2_: NaCl Conc. (mg. L^−1^)	2500	5000	7500	10,000	12,500
X_3_: Current intensity (A)	0.06	0.12	0.18	0.24	0.3
X_4_: Electrolysis time (min)	7.5	15	22.5	30	37.5

### Experimental set-up and procedure

**Fig. 2:**
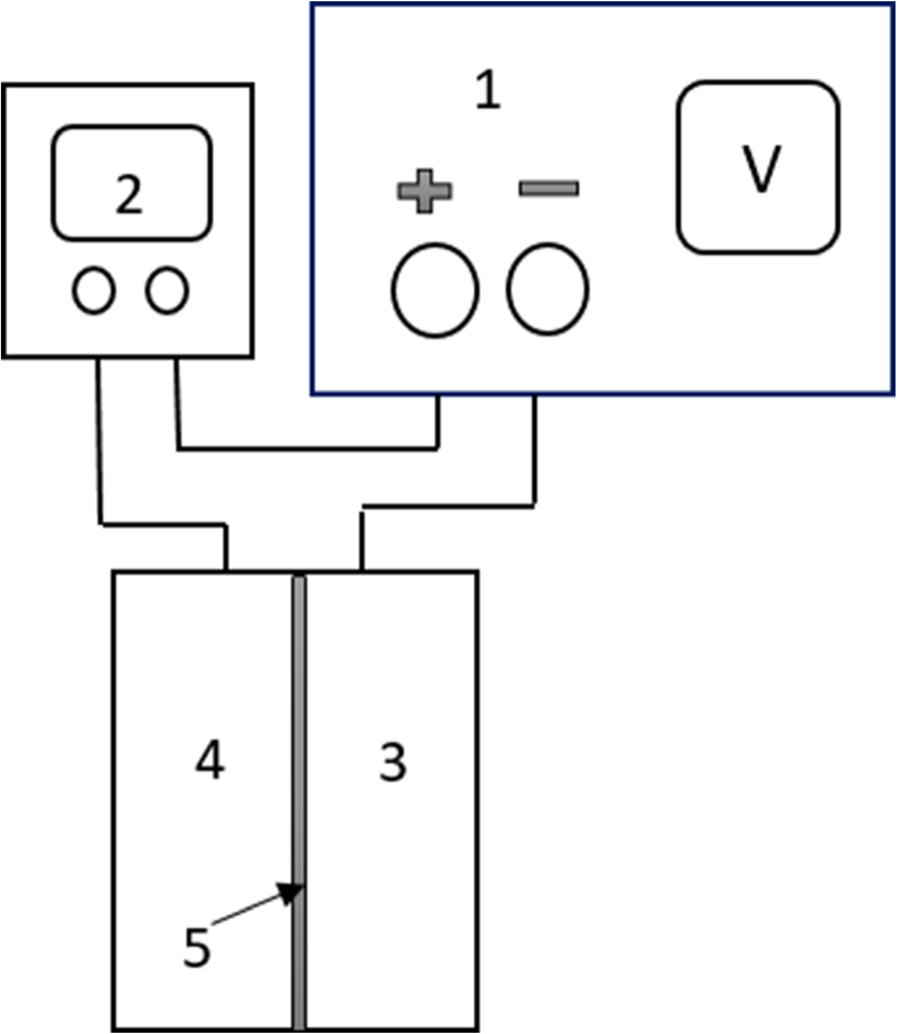
Scheme of the experimental set up: (1) DC power supply, (2) multimeter, (3) cathode chamber, (4) anode chamber, and (5) cellulosic separator

Figure 2 gives the schematic of the electrochemical cell consisted of a rectangular reactor coupled with the power supply and multimeter. The hold-up container was a Plexiglass vessel in which two electrodes were placed close to the both sides of the separator. The separator was made of cellulose fibers (cotton) located in the middle of the reactor to divide it into two distinct equal parts. A rod of graphite was used as the anode electrode while a sheet of stainless steel was applied as the cathode electrode. An adjustable laboratory DC power supply was used to provide the electrical energy needed to operate the system. The test solutions were prepared from the stock solution and completely mixed using a magnetic stirrer before experiments. Treatment was carried out in a batch system with a net working volume of 200 mL. In each experiment, sampling was done from both parts of the reactor at the time determined by CCD, and the pH of the samples was neutralized by dilute solutions of H_2_SO_4_ and NaOH. The acidic pH of the anolyte contents was due to water hydrolysis at the anode which produced H^+^ ions and the alkaline pH of the catholyte was due to the H_2_ and/or O_2_ reduction reactions that consume H^+^ and generate OH^−^ at the cathode zone [32]. To measure the remained concentration of dye, the Milton Roy Company Spectronic 20 Spectrophotometer (UV–VIS) was applied at 530 nm.

### Analytical methods

To determine the dye concentration, at first a calibration curve (R^2^ = 0.9999) was drawn based on the absorbance levels corresponding to samples of known concentration. Then, the following expression (Eq. 14) was used to calculate the final dye concentration:

14 $$ \left(\left(48.416.\mathrm{A}\right)\ \hbox{--}\ 0.1718\right)\mathrm{d}\mathrm{f} $$

Where A is the absorbance of the solution and df shows the dilution factor. Then, the removal efficiency was calculated according to Eq. (15):

15 $$ \frac{C_{in}-{C}_f}{C_{in}}\times 100 $$

Where the initial and final color concentrations are shown by C_in_ and C_f_, respectively. The average potential of the cell (V) during the electrolysis was recorded to calculate the energy consumption according to Eq. (16):

16 $$ E C = \left(\frac{ V AT}{1000{V}_S}\right) $$

Where EC shows the energy consumption per volume of treated wastewater (Kwhm^−3^), T is the time of electrolysis (h), A and V_s_ are the current intensity, and sample volume (m^3^), respectively. Finally, the obtained data were analyzed in Design Expert 7 program.

## Results and Discussion

In the current study, a set of electrochemical batch experiments were performed in a two-compartment reactor divided by cellulosic separator to study the effectiveness of EO and ER using graphite and stainless steel electrodes, respectively, in the removal of RR120 from synthetic textile effluents. Cellulose was applied for cell separation in the present research because of its great benefits such as its natural abundance which subsequently results in the lower cost of operation. Its physical characteristics such as porosity and permeability cause satisfactory separation between the contents of anolyte and catholyte chambers. Cellulose ability for retaining a layer of water in its structure allows establishing electric flux between the contents of separated compartments [26].

In this work, the effect of the operational parameters including initial dye concentration, electrolyte concentration, current intensity, and electrolysis time on the RR120 removal efficiency was studied within a specified range. Accordingly, the effect of initial RR120 concentration on its removal efficiency was studied in the range between 100 mg. L^−1^ and 500 mg. L^−1^. Cardoso et al. [33] investigated the removal of RR120 from aqueous effluents through adsorption process within the range of 50–1200 mg. L^−1^. Tehrani-Bagha and Amini [34] studied the effectiveness of UV-Enhanced Ozonation for the treatment of simulated dyebath effluents containing 200 mg. L^−1^ and 800 mg. L^−1^ of RR120. Another factor affecting the RR120 removal rate was NaCl concentration that was studied from 2500 to 12,500 mg. L^−1^. The concentration of this electrolyte in the real textile wastewater has been reported from 5000 to 12,000 mg. L^−1^ [29]. The impact of current intensity on the treatment efficiency in this study was assessed in the range between 0.06 A and 0.3 A that was close to the range of 0.1–0.35 A in a study conducted by Ghalib [35] for electrochemical removal of direct blue dye from textile wastewater. It was decided to apply electrical current at such low intensities because it is known that graphite electrodes have small values of overvoltage for oxygen evolution, indicating their effective performance for pollutant oxidation only at very low current intensities [1]. In the present work, the electrolysis time was studied at five points from 7.5 to 37.5 min. In the Zaviska et al. [36] study for atrazine removal using EO process, the effect of treatment time was assessed at two levels: 10 min and 40 min. The time of electrolysis varied from 5–20 min in Kariyajjanavar et al. [4] study for electrochemical degradation of reactive azo dyes from aqueous solutions using graphite electrodes.

### Regression models and ANOVA

In this study, a total of 30 runs were performed according to the CCD suggestions to assess the relationship between each response and four independent variables. For this purpose, a mathematical equation was developed for every response in RSM to study the behavior of the system as a function of RR120 concentration (x_1_), NaCl concentration (*x*_2_), current intensity (x_3_), and electrolysis time (x_4_). After removing model terms which were not statistically significant because of the Prob > F > 0.05, each equation was achieved as a sum of a constant value, and main, interaction, and quadratic effects in the model. The modified models are shown in the following:

17 $$ {Y}_1\left( RR120\  removal\  in\  anolyte\  chamber\right)\%=99.32-2.98{x}_1+2.22{x}_2+12.06{x}_3+9.94{x}_4+4.34{x}_1{x}_3+2.60{x}_1{x}_4-2.80{x}_2{x}_3-9.05{x}_3{x}_4-6.74{x}_3^2-5.62{x}_4^2 $$

18 $$ {Y}_2\left( RR120\  removal\  in\  catholyte\  chamber\right)\%=22.34-6.72{x}_1+4.72{x}_3+8.05{x}_4 $$

19 $$ {Y}_3\left( Energy\  consumption\right) k w h{m}^{-3}=1.65+0.73{x}_3+0.51{x}_4+0.22{x}_3{x}_4 $$

After screening the models to exclude insignificant effects, the experimental data were analyzed using ANOVA to check the adequacy of the models. Based on F-test results which are given in Table 3, the quadratic model for Y_1_, the linear model for Y_2_, and the 2FI model for Y_3_ were all highly significant. The F-values were 49.24, 20.48, and 236.49 for the functions corresponding to Y_1_, Y_2_, and Y_3_, respectively. The chance of achieving these large values of F due to error is only 0.01%. Furthermore, the R^2^ coefficient was 0.963 for Y_1_, 0.703 for Y_2_, and 0.964 for Y_3_. With respect to R^2^ value which measures the proportion of total variations in the dependent variable that can be explained by the model predicators [37], the models predictions were in good agreement with the experimental data. Since some of the variables were excluded from the regression model in the modification process, the R^2^ index was calculated using the variables retained in the model and was named the adjusted R^2^. The difference between this index and the R-squared predicted by the model must be a number lower than 0.2 to ensure well data fitting by the developed model [31]. The disagreement between the adjusted and predicted R^2^-values for all models was less than 0.09 (see under Table 3). The models precision was adequate because of the signal/noise ratio more than 4 in all cases. Although graphs of normal % probability and studentized residuals are not shown, regarding the fairly straight lines of these graphs, the distribution of data was normal for all responses.

**Table 3:** Statistical indices obtained from the ANOVA for regression models

Source	Sum of squares	Degrees of freedom	Mean square	*F* value	*P > F*
In the anolyte compartment ^a^					
Model	10010.49	10	1001.05	49.24	<0.0001
Residual	386.23	19	20.33	Na	Na
Lack of fit	385.87	14	27.56	381.01	<0.0001
Pure Error	0.36	5	0.072	Na	Na
In the catholyte compartment ^b^					
Model	3173.34	3	1057.78	20.48	<0.0001
Residual	1342.74	26	51.64	Na	Na
Lack of fit	1162.51	21	55.36	1.54	0.3362
Pure Error	180.23	5	36.05	Na	Na
Energy consumption ^c^					
Model	19.81	3	6.60	236.49	<0.0001
Residual	0.73	26	0.028	Na	Na
Lack of fit	0.53	21	0.025	0.64	0.7861
Pure Error	0.2	5	0.039	Na	Na

*Na* Not applicable

^a^ R^2^ = 0.963, R_adj_^2^ = 0.943, R_pred_^2^ = 0.879, adequate precision = 23.549

^b^ R^2^ = 0.703, R_adj_^2^ = 0.668, R_pred_^2^ = 0.579, adequate precision = 14.855

^c^ R^2^ = 0.964, R_adj_^2^ = 0.961, R_pred_^2^ = 0.952, adequate precision = 47.801

### Dye removal efficiency in anodic (oxidative) cell

**Fig. 3:**
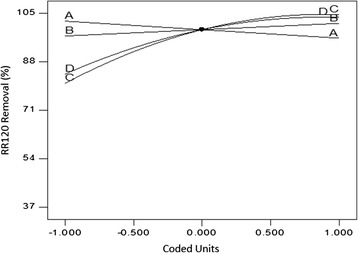
Perturbation plot of RR120 removal via EO process as a function of A (Dye conc.), B (NaCl conc.), C (Current intensity), and D (Electrolysis time)

**Fig. 4:**
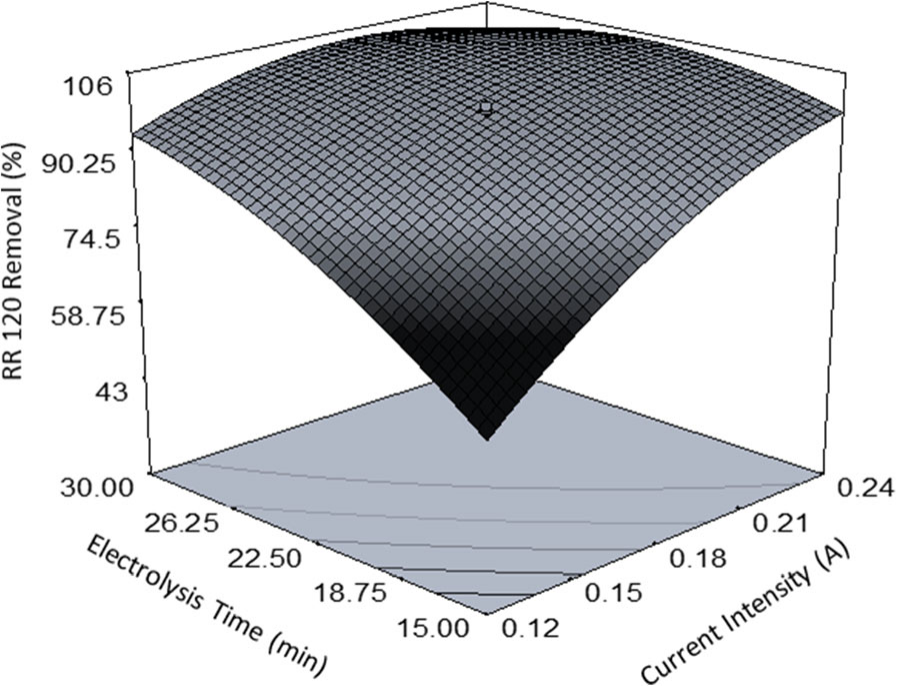
3D surface plot to illustrate dye removal as a function of simultaneous effect of reaction time and current intensity in the anodic cell

The first part of Eq. (17) shows 99.32% removal efficiency for RR120 from the anolyte content when all terms in the second part of the equation are fixed at their central values. The magnitude of the coefficient devoted to each term and the corresponding positive or negative sign determines the variations that may occur in the RR120 removal rate when the levels of the experiment factors in the of the equation change. Equation 17 indicates that the positive coefficient (+12.06) related to the current intensity factor had the highest value among different coefficients; thus, this factor created the most meaningful effect on the response. The next rank was allocated to the effect of contact time with the coefficient of +9.94. According to Table 4 which represents the experimental results as a function of various levels of independent parameters, there was a direct relationship between the two aforementioned variables and the study response, which can also be concluded from the perturbation plot of Fig. 3. With regard to the literature review, current intensity has been the most important factor affecting the performance of EO process in lab scale [2, 6]. Curve C in the Fig. 3 shows a steep increase in the dye removal rate from the level −1 to the central level of current intensity, while this rate increases gradually up to the level +1 and then stops. This behavior has been indicated by the significant quadratic effect devoted to the applied current in Eq. 17. This negative second order effect can be explained by considering the nature of the graphite as it has low values of overpotential for O_2_ evolution. It is known that in higher current intensities, the parasite nonoxidizing reaction of O_2_ evolution is a dominant mechanism which causes a significant reduction in current efficiency. Thus, applying low current intensities can be effective for oxidation of pollutants on this anode [1]. In addition to the discussed first and second order effects of the current intensity, this factor also showed the most important combined effect on the response in the interaction with the electrolysis time parameter. 3D surface plot of Fig. 4 shows this interaction. As can be seen, instead of applying high levels of both factors to reach a favorable removal efficiency, electrolysis can be performed at lower intensities and higher reaction times or vice versa or at moderate levels of both parameters to yield the same removal percentage. The negative sign of the respective quadratic effect confirms this concept.

**Table 4:** Experimental conditions determined by CCD and the observed results

Run No.	Independent variables				Dependent variables		
	RR120 Conc. (mg. L^−1^)	NaCl Conc. (mg. L^−1^)	Current intensity (A)	Electrolysis time (min)	Decolorization efficiency via EO (%)	Decolorization efficiency via ER (%)	Energy consumption (kwhm^−3^)
1	300	7500	0.06	22.5	43.4	11.8	0.365
2	400	10,000	0.24	15	99	13.26	1.44
3	400	5000	0.12	15	37.2	9.29	0.525
4	300	7500	0.18	22.5	98.5	27	1.333
5	300	7500	0.18	22.5	99.2	31.2	1.50
6	400	10,000	0.12	15	52	2.7	0.577
7	200	5000	0.24	15	99.2	22.76	1.71
8	300	12,500	0.18	22.5	99.2	20.65	1.636
9	200	10,000	0.24	15	99.1	22.8	1.65
10	500	7500	0.18	22.5	98.4	17.26	1.603
11	200	10,000	0.24	30	98.58	48.56	2.64
12	400	5000	0.24	30	98.45	16.94	3.12
13	100	7500	0.18	22.5	98.72	52.6	1.67
14	400	10,000	0.24	30	99.34	34.5	3.24
15	300	7500	0.18	22.5	99.17	22.39	1.62
16	200	5000	0.12	15	64.65	10	0.675
17	300	7500	0.18	7.5	52	14.49	0.534
18	200	5000	0.24	30	98.2	38.85	3.39
19	300	7500	0.18	22.5	99.19	18	1.636
20	300	7500	0.3	22.5	98.88	30	3.26
21	300	7500	0.18	22.5	99	22.11	1.755
22	200	10,000	0.12	15	93.3	16.14	0.637
23	300	7500	0.18	22.5	99	14.49	1.906
24	400	10,000	0.12	30	98.48	16.31	1.17
25	400	5000	0.12	30	82.54	20.87	1.08
26	200	10,000	0.12	30	99.34	22.32	1.275
27	300	2500	0.18	22.5	97.73	16.29	1.84
28	400	5000	0.24	15	97.59	2.18	1.725
29	200	5000	0.12	30	97.37	25.19	1.275
30	300	7500	0.18	37.5	99.32	48.84	2.56

Equation 17 also shows the sensitivity of decolorization efficiency to the effect of RR120 concentration. It can be observed in Fig. 3 that when the dye concentration increased from 200 to 400 mg. L^−1^, the removal percentage reduced to 6%. This result is in agreement with results already reported by Körbahti et al. who studied electrochemical decolorization of textile dyes [6]. As can be seen from curve B in the perturbation plot of Fig. 3, the addition of NaCl as electrolyte into the working solution resulted in a better removal performance which can be attributed to the increased cell conductivity and generation of powerful oxidizing agents such as Cl_2_ and HOCl. The former will increase the ionic transfer and current intensity at a given operating voltage which provides more chance for the latter. The acidic pH of the anodic cell is in favor of electrochemical degradation of azo dyes since it causes chloride reduction to chlorine gas and further to hypochlorous acid which is known for its high potential of oxidation [4]. The anodic degradation of the RR120 can be explained by the action of the chemisorbed hydroxyl radicals and also the active chlorine species. The reactions involved in the formation of chlorine based oxidants are completely expressed in the introduction section, but regarding the role of chemically adsorbed hydroxyl radical or M(^•^OH) it must be considered that the strong interaction of the electrode surface with the ^•^OH does not allow its direct reaction with organics and instead, a superoxide (MO) is formed according to Eq. (20). MO further acts as a mediator in the oxidation of organics by reaction presented in Eq. (21):

20 $$ \mathrm{M}\left({}^{\bullet}\mathrm{O}\mathrm{H}\right)\ \to\ \mathrm{M}\mathrm{O} + {\mathrm{H}}^{+} + {\mathrm{e}}^{\hbox{-} } $$

21 $$ \mathrm{M}\mathrm{O} + \mathrm{R}\ \to\ \mathrm{M} + \mathrm{R}\mathrm{O} $$

As previously discussed in the introduction section, achieving to complete mineralization via electrochemical combustion is unexpected when the used anode is an active one. Instead, the electrochemical conversion of organics into reaction intermediates is happened [24]. This was proved through the Fourier transform infrared spectroscopy (FTIR) analysis (data not shown). The band at 3447.95 cm^−1^ corresponds to the N-H stretching vibration [10], while the peak appeared at 1717.62 cm^−1^ belongs to the carbonyl region and can be ascribed to -C = O stretching vibration [38]. The appearance of peak at 1594.80 cm^−1^ which shows the -N-H bending mode suggests the formation of the amino group by the cleavage of azo bond [39].

### Dye removal efficiency in cathodic (reductive) cell

**Fig. 5:**
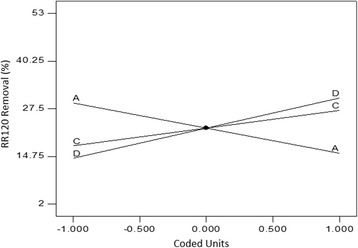
Perturbation plot of RR120 removal via ER process as a function of A (Dye conc.), C (Current intensity), and D (Electrolysis time)

With respect to Eq. (18), 22.34% of removal efficiency was observed for RR120 via reductive pathway, which was independent of any factor and interaction of factors. The model indicates the direct relationship of RR120 removal with the main effects of applied current (+4.72) and time (+8.05). On the contrary, the removal performance was negatively associated with RR120 initial concentration with respect to the coefficient of −6.72. This effect can also be seen in Table 4, where the maximum removal efficiency of 52% was observed for the solution containing 100 mg. L^−1^ of RR120 as the lowest examined concentration. The line A in the perturbation plot of Fig. 5 suggests that when the concentration of RR120 was doubled in the solution, 14% reduction occurred in its removal efficiency. The middle line in the Fig. 5 corresponds to the effect of current intensity which caused 10.6% higher removal efficiency when its level increased from 0.12 A to 0.24 A. The effect of electrolysis time was more pronounced when it increased from 15 min to 30 min, leading to 16% higher removal performance. Regardless of the parameters and the effect of different levels of them on the response, the total performance of reductive cell used in the present research for RR120 removal was low. This is in agreement with the study conducted by Carneiro et al. [40] who achieved only 37% RB4 removal via ER process.

Two possible pathways can be proposed for degradation of RR120 in the cathodic cell. The dye may be adsorbed on the surface of stainless steel and then the direct cathodic electron transfer may occur [41] according to the Eqs. (11) and (12) presented in the introduction section. For the latter mechanism, we can refer to the role of hydrogen. When stainless steel is used as cathode, the chemisorbed hydrogen is generated at the electrode surface by electrolysis of water [2] according to the Eq. (13) which can then participate in decolorization. At basic pH of the catholyte compartment, hypochlorite ions are dominant species in the bulk, resulting in the cleavage of azo bond. Oxidation of amid group can lead to the generation of carboxylic derivatives and thus weak acidic condition. The nitrogen in the azo bond is reduced by accepting hydrogen, the double bond transforms to single bond and then to amine. The weak acidic condition encourages the amine compounds to accept proton and as a result, it can be adsorbed onto the negative charged sites of the cathode [1].

### Energy consumption

**Fig. 6:**
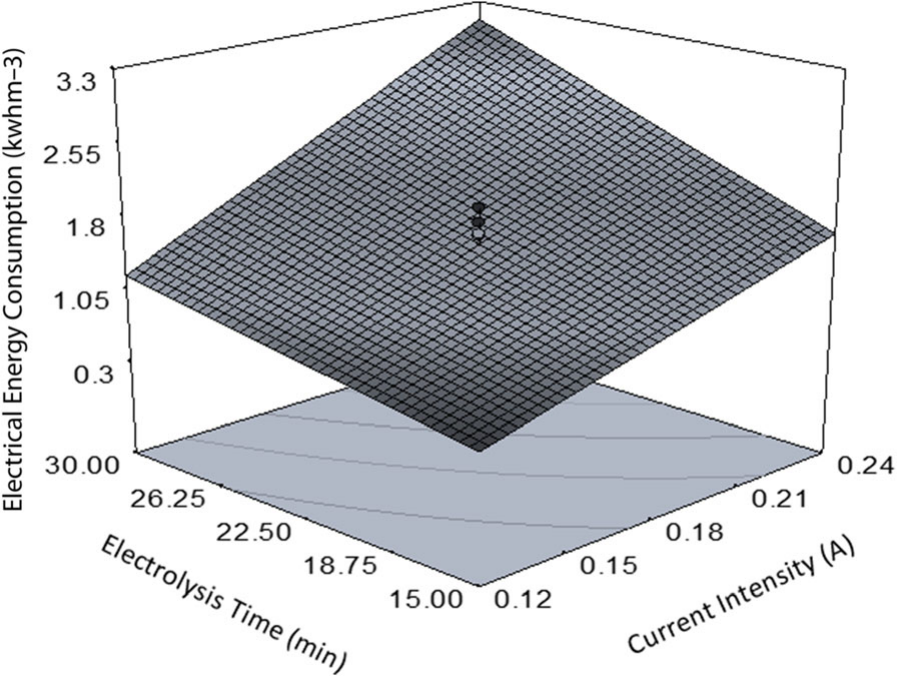
3D surface plot for exhibition the simultaneous effect of current intensity and electrolysis time on power consumption

With respect to Eq. (19), the average amount of consumed energy for the removal of RR120 in the designed electrochemical cell was 1.65 kwhm^−3^ that was remarkably lower than consumed energy in similar studies [4, 5, 11]. Furthermore, it was proved that energy consumption is proportional to the current intensity and electrolysis time and also the combined effect of these two factors. A change in the applied current and time from level −1 to +1 resulted in more energy consumption as 1.46 kwhm^−3^ and 1.02 kwhm^−3^, respectively. Hence, the energy consumption was more affected by the variations occurred in the level of applied current in comparison to the reaction time which can be attributed to the increased oxygen and hydrogen evolution reaction in the anodic and cathodic cell, respectively, at higher current intensities [4]. Figure 6 indicates current intensity interaction with electrolysis time on energy consumption rate while the two other factors were constant at the central level. As obviously seen in the graph, there was a synergistic effect between the parameters and the response. According to RSM prediction, 3 kwhm^−3^ of electrical energy will be consumed if the solution is treated at the level +1 of both factors.

### Optimization

In the optimization process of RR120 removal, the criteria goal was selected “in range” for all the independent variables while it was desired as “maximize” for removal efficiency and as “minimize” for power consumption. The optimum values of parameters proposed in first solution were: initial RR120 concentration (200 mg. L^−1^), NaCl concentration (7914.29 mg. L^−1^), current intensity (0.12 A), and reaction time (30 min), leading to 99.44 and 32.38% of RR120 removal via EO and ER mechanisms, respectively, with consuming 1.21 kwhm^−3^ of electrical energy. According to the confirmation study carried out in optimal conditions, the removal performance via EO was obtained as 96% that was very close to the predicted value. The removal efficiency in the sample drawn from the catholyte compartment was 22%. This value also located in the predicted interval (PI low: 16.49% and PI high: 48.28%). In addition, 1.3 kwhm^−3^ of electrical energy was consumed in the conducted experiment that was also was in the PI. Considering the low performance of cathodic degradation, chemical oxygen demand (COD) analysis was just conducted on the sample taken out from the anolyte compartment. The analysis revealed that only 17.56% of COD was removed during the electrolysis. However, achieving such a low efficiency in COD removal with respect to the electrolysis performed in a short time (30 min) at low current intensity (0.12 A) was not unexpected. Rajkumar and Kim achieved 73.5% COD reduction for a mixture of reactive dyes at a concentration of 200 mg. L^−1^ after 120 min electrolysis time using 2 A of current intensity [29]. Although the levels of operating parameters in the current work satisfied the goal of the study, i.e., cost-effective removal of RR120 under optimal conditions, given the importance of mineralization, it is suggested for further investigation to try longer electrolysis times and higher electrical currents to reach a remarkable COD removal.

## Conclusion

This investigation assessed the performance of electro-oxidation and electro-reduction pathways by means of graphite and stainless steel electrodes in a two-compartment reactor divided by cellulosic separator in the removal of RR120 dye from synthetic textile effluent. Based on the results, some conclusions are drawn as follows:

i. Anodic oxidation using graphite electrode was successfully applied for decolorization of strongly colored effluent and gave ≥90% removal efficiency in four fifth of the experiments.
ii. The reductive pathway using stainless steel failed to achieve a satisfactory removal rate. The maximum RR120 removal rate in cathodic cell was 52% obtained for the most dilute solution.
iii. The average amount of electrical energy consumption 1.65 kwhm^−3^ was much less than the corresponding values in similar studies, mainly due to the low levels of applied current and time of electrolysis.
iv. 96% dye removal efficiency was obtained in the anodic cell under the optimized operating conditions of 7914 mg L^−1^ NaCl, 0.12 A current intensity, and 30 min reaction time for a solution containing 200 mg L^−1^ of RR120 concentration.
v. The RR120 degradation was removed due to electrochemical conversion that caused formation of intermediate products. With respect to the low reduction of COD, higher levels of current intensity and electrolysis time should be tried to provide the opportunity for dye intermediates to be converted to CO_2_ and H_2_O.

## Abbreviations

ANOVA: Analysis of variance
AO: Anodic oxidation
AOPs: Advanced oxidation processes
CCD: Central composite design
COD: Chemical oxygen demand
DDW: Double distilled water
EAOPs: Electrochemical advanced oxidation processes
EC: Electrocoagulation
EF: Electro-fenton
EO: Electro-oxidation
ER: Electro-reduction
FTIR: Fourier transform infrared spectroscopy
H_2_SO_4_: Sulfuric acid
NaCl: Sodium chloride
NaOH: Sodium hydroxide
PI: Prediction interval
RR120: Reactive red 120
RSM: Response surface methodology
